# Radiomics in esophageal and gastric cancer

**DOI:** 10.1007/s00261-018-1724-8

**Published:** 2019-06-01

**Authors:** Bert-Ram Sah, Kasia Owczarczyk, Musib Siddique, Gary J. R. Cook, Vicky Goh

**Affiliations:** 1 Department of Cancer Imaging, School of Biomedical Engineering and Imaging Sciences, King’s College London, London, UK; 2 King’s College London and Guy’s and St Thomas’ PET Centre, St Thomas’ Hospital, London, UK; 3 Department of Radiology, Guy’s St Thomas’ Hospitals NHS Foundation Trust, London, UK; 4 Radiology, Level 1, Lambeth Wing, St Thomas’ Hospital, Westminster Bridge Road, London SE1 7EH, UK

**Keywords:** Esophageal cancer, Esophagogastric junction cancer, Radiomics, Computed tomography, Magnetic resonance imaging, Positron emission tomography

## Abstract

Esophageal, esophago-gastric, and gastric cancers are major causes of cancer morbidity and cancer death. For patients with potentially resectable disease, multimodality treatment is recommended as it provides the best chance of survival. However, quality of life may be adversely affected by therapy, and with a wide variation in outcome despite multi-modality therapy, there is a clear need to improve patient stratification. Radiomic approaches provide an opportunity to improve tumor phenotyping. In this review we assess the evidence to date and discuss how these approaches could improve outcome in esophageal, esophago-gastric, and gastric cancer.

## The need for better patient stratification

Esophageal or esophago-gastric cancer (456,000 new cases annually) and gastric cancer (952,000 new cases annually) are leading causes of cancer deaths worldwide [1]. Above 50% of presenting patients are diagnosed with stage IV disease, precluding curative treatment. For patients with early stage disease, surgery, often combined with neoadjuvant chemotherapy or chemoradiotherapy, offers the best chance of cure [2–7].

As an example, data from the OEO2 and MAGIC trials for esophageal and esophago-gastric cancer have shown a 6% [3] and 13% [4] improvement in 5-year overall-survival, respectively. Trimodality therapy for esophageal and esophago-gastric cancer combining neoadjuvant chemo- and radiation-therapy in addition to surgery may also be superior to neoadjuvant chemotherapy alone in a selected patient population [8]. The CROSS trial [6] comparing neoadjuvant chemoradiotherapy plus surgery with surgery alone in patients with esophageal and esophago-gastric cancer showed a superior overall-survival of 49 vs. 24 months, hazard ratio 0.657, p = 0.003, and a pathological complete response rate of 29%, for patients with multi-modality treatment with no increase in surgical mortality (4% in surgery and trimodality groups, respectively).

**Fig. 1:**
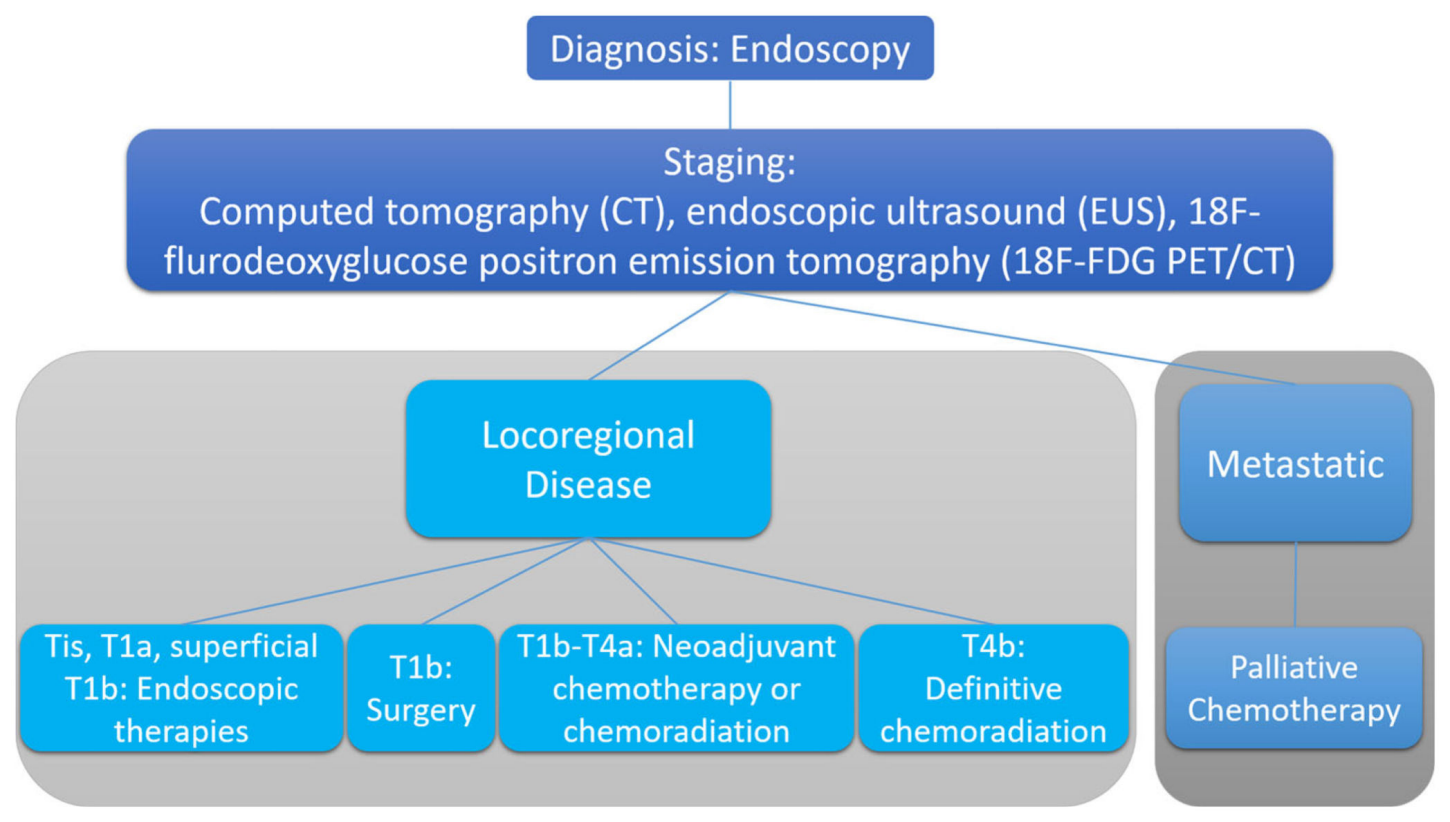
Typical pathways for the management of patients with newly diagnosed esophageal and esophago-gastric cancer.

**Fig. 2:**
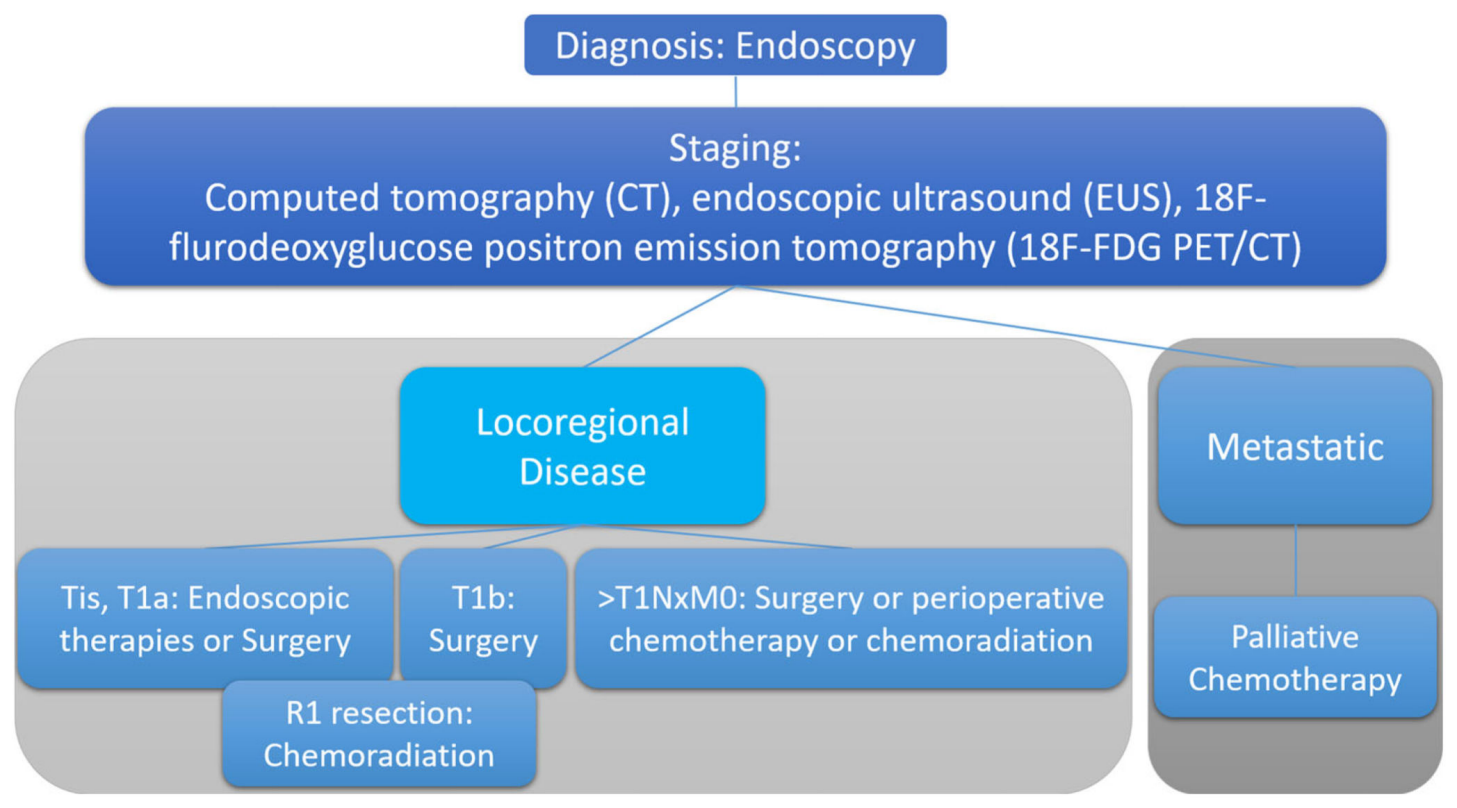
Typical pathways for the management of patients with newly diagnosed gastric cancer.

A recent systematic review and meta-analysis of neoadjuvant chemotherapy in patients with gastric cancer has also found improved 3-year survival rates (relative risk 1.30; 95% CI 1.06–1.59, *p* < 0.01) [9]. Typical management pathways are shown in Figs. 1 and 2 for esophageal, esophago-gastric, and gastric cancer, respectively. Nevertheless overall-survival remains poor despite these improvements in patient care.

Recent genomic analyses have highlighted the genetic heterogeneity present in esophageal, esophago-gastric [10], and gastric cancer [11, 12] as an underlying cause for the differences in outcome and heterogeneity of response to therapy. Quality of life also remains poor for many patients post-surgery, taking up to 3 years to return to pre-therapy levels in patients undergoing esophageal resection [13]. Better patient stratification remains a key challenge for patients with upper gastrointestinal tract cancers.

## The imaging pathway at staging

For esophageal and esophago-gastric cancer, contrast-enhanced computed tomography (CT) remains the most commonly performed first step in staging due to the high prevalence of metastatic disease at presentation [14–16]. For patients being considered for a curative pathway, endoscopic ultrasound (EUS) and 18F-fluorodeoxyglucose positron emission tomography/CT (18F-FDG PET/CT) are performed due to the high sensitivity and specificity of EUS for local tumor and nodal staging; and 18F-FDG PET for distant metastases [17–19]. This aims to reduce the futile surgery rate.

For gastric cancer, initial staging is again by contrast-enhanced CT. If curative treatment is being considered, the use of endoscopic ultrasound (EUS) is helpful in determining the proximal and distal extent of the tumor, whereas 18F-FDG PET/CT has been shown to improve staging by detecting involved lymph nodes and metastatic disease, although it can be less accurate in mucinous and diffuse tumors [20].

In esophageal cancer, PET has the potential to change management in up to a third of patients [21, 22], and is often incorporated into radiotherapy planning pathway [23, 24]. The American College of Surgeons Oncology Group reported sensitivity and specificity of 18F-FDG-PET/CT scans to be 79% and 95%, respectively [18].

Magnetic resonance imaging (MRI) is currently not recommended for the routine imaging of esophageal or gastric cancer. However, with the recent advent of hybrid PET/MRI systems in clinical practice, there has been growing interest in MRI’s ability as an assessment tool. MRI provides excellent soft-tissue contrast, and may demonstrate the esophageal wall layers and adjacent nodes. Physiological sequences (e.g., diffusion-weighted MRI) may also be included as part of the protocol. An initial staging 18F-FDG PET/MRI study with a pathology gold standard has been promising for nodal assessment with reported accuracy of 83% compared to 75% and 50% for EUS and CT, respectively [25].

## A role for radiomics?

Radiomic approaches are showing promise for patient stratification. Radiomics exploit the data performed as part of the clinical management pathway. In terms of imaging, a number of parameters may be extracted and combined including standard descriptors (e.g., size, morphology, TNM (tumor, node, metastasis) stage); qualitative, semi-quantitative, or quantitative physiological parameters (e.g., contrast enhancement, diffusion characteristics, tracer uptake); and additional agnostic features which are otherwise ‘invisible’, with bioinformatic approaches. Of these, texture-based features have been investigated most commonly to date. Table 1 highlights some features that have been investigated in studies.

**Fig. 3:**
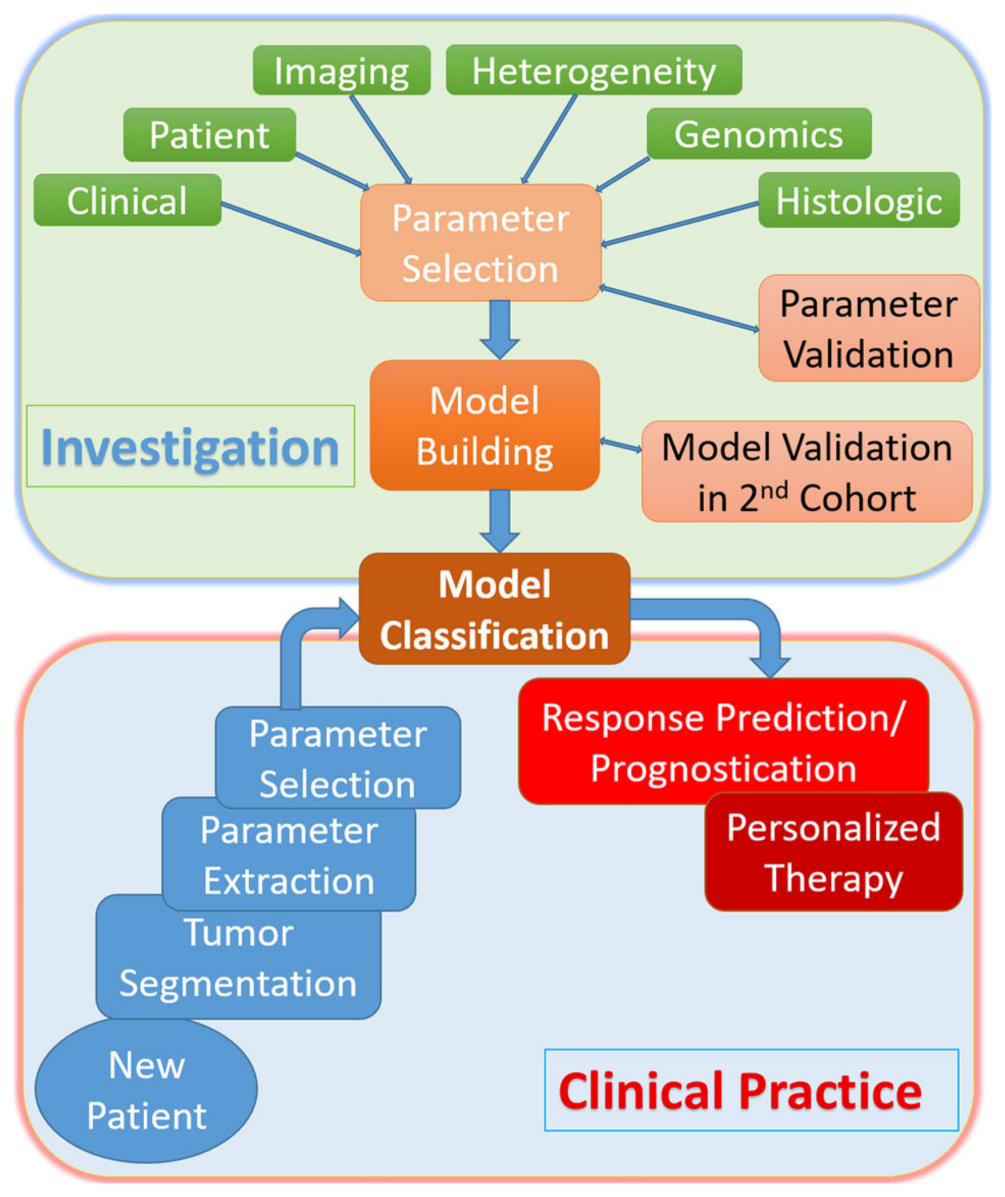
Schema demonstrating typical radiomics pipeline.

**Fig. 4:**
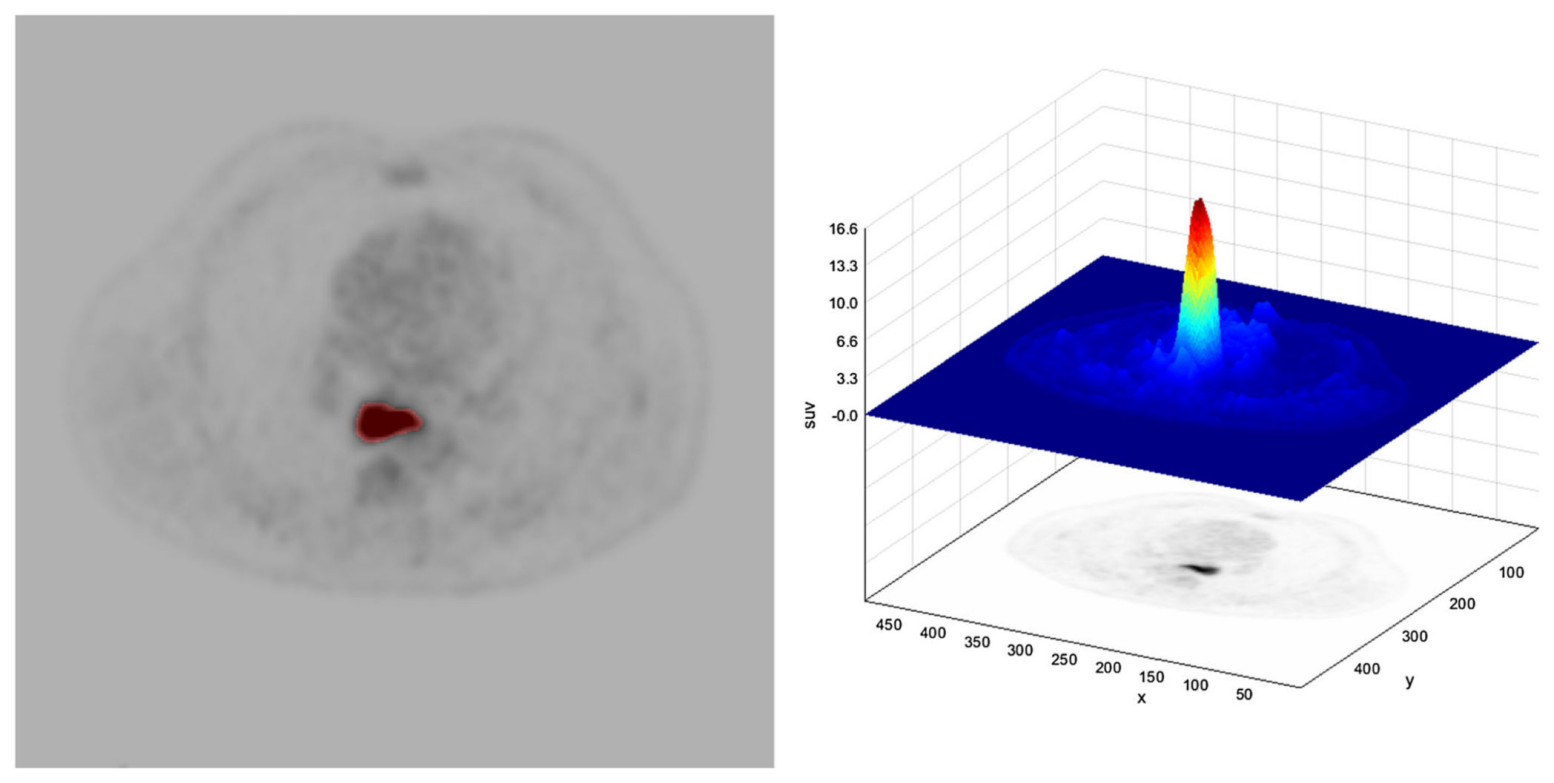
Example of tumor segmentation for extraction of radiomic features from an axial PET image. In the right image the corresponding standardized uptake values for the region-of-interest is displayed.

Radiomic signatures provide additional information predictive of underlying tumor biology and behavior. These signatures can be used alone or with other patient-related data (e.g., pathological data; genomic data) to improve tumor phenotyping, treatment response prediction and prognosis. Radiomic signatures may be obtained for all cross-sectional imaging modalities, including CT, PET, and MRI. figure 3 illustrates a typical radiomics pipeline.figure 4 demonstrates the process of tumor segmentation for a 18F-FDG PET image with a corresponding plot of standardized uptake value for the tumor. Initial studies in esophageal, esophago-gastric, and gastric cancer have shown promise for patient care.

## 18F-FDG PET radiomics

Nine 18F-FDG PET studies have been performed in esophageal and esophago-gastric cancer and are summarized in Table 2. As yet no studies have been performed for gastric cancer. Studies to date have focused on the prediction of response or prognosis in comparison to standard practice. Studies have found that various first, second and high-order features have been contributory to the assessment of response, differentiating between responders and non-responders (with greater heterogeneity in non-responders), as well as being predictive of complete response. Performance has been better than conventional parameters alone. Prognostication data remain conflicting.

In greater detail, five studies have investigated the prediction of response to therapy alone (*n* = 2); prognosis alone (*n* = 1) and the prediction of response to therapy and prognosis (*n* = 2) from pre-therapy imaging. One of the earliest studies by Tixier et al. showed in 41 patients that gray-level co-occurrence matrix (GLCM) homogeneity, GLCM entropy, gray-level size-zone matrix (GLSZM) size-zone variability and run length matrix (RLM) intensity variability differentiated non-responders, partial-responders, and complete-responders with sensitivities of 76%–92% [26]. Beukinga et al. showed in 97 patients that a clinical model including PET-derived gray-level run length (GLRL) long run low gray level emphasis and CT-derived run percentage had a higher area under the receiver operator curve (AUROC) compared to maximum standardized uptake value (SUV_max_) in predicting therapy response [27].

In a study of 52 patients with squamous cancers, Nakajo et al. found that 18F-FDG PET/CT GLSZM intensity variability, and GLSZM size-zone variability, as well as standard volumetric parameters, such as metabolic tumor volume (MTV) and total lesion glycolysis (TLG), were predictors of tumor response but not of progression free or overall-survival [28]. Non-responders showed significantly higher intensity variability and size-zone variability. Similarly, in a study of 65 patients Paul et al. found that a model incorporating GLCM homogeneity was a predictor of response (with an AUROC value of 0.823) but not of survival [29]. However, in a larger study with 403 patients, Foley et al. found that total lesion glycolysis, histogram energy and kurtosis were independently associated with overall-survival [30].

Four studies have assessed pre- and post-therapy 18F-FDG PET imaging. An initial study by Tan et al. found in 20 patients that 2 SUV_mean_ parameters, SUV_mean_ decline and SUV_mean_ skewness, and 3 texture features GLCM inertia, GLCM correlation, and GLCM cluster prominence, were significant predictors of complete response with an AUROC of 0.76 [31]. In 217 patients with adenocarcinoma, Van Rossum et al. developed a prediction model which included change in run length matrix (RLM) run percentage, change in GLCM entropy, and post–chemoradiation roundness, and increased the corrected c-index (concordance-index, comparable to AUROC) from 0.67 to 0.77, compared to the clinical model alone [32]. Yip et al. found that a change in run length and size-zone matrix differentiated responders from non-responders [33]. More recently, Beukinga et al. found that clinical T-staging combined with post-chemoradiotherapy ^18^F-FDG PET orderliness provided high discriminatory accuracy in predicting pathologic complete response compared to clinical variables or SUV_max_ alone [34].

## CT radiomics

Nine studies have investigated the ability of CT-derived heterogeneity parameters for classification, prediction of response and overall-survival in patients with esophageal or gastric cancer. Three studies have been performed for esophageal cancer in terms of prediction of response or prognosis (Table 3). These have found that greater heterogeneity is present in non-responders and those with poorer outcome.

The largest study in 49 patients found that histogram skewness, histogram kurtosis, GLSZM long-zone emphasis, and 2 Gabor transformed parameters MSA-54 and MSE-54, discriminated non-responders from responders using an artificial neural network-derived prediction model [35]. The two remaining studies have assessed prognostication. Ganeshan et al. found that lower histogram uniformity (with Gaussian filtration) from unenhanced CT images before start of treatment was an independent predictor for poorer overall-survival [36].Yip et al. analyzed contrast-enhanced images of 36 patients before and after treatment and found a significant decrease in histogram entropy and increase in uniformity (with Gaussian filtration) between the two time points. Higher post treatment entropy was associated with poorer overall-survival [37].

For gastric cancer (Table 4), three studies have assessed the potential of radiomic approaches for classification. Studies have found that first and second-order analysis in the contrast-enhanced images may help in differentiation of lymphoma from gastrointestinal stromal cancer [38] or adenocarcinoma [39]. Another study in 107 patients found that arterial phase standard deviation and entropy were correlated with poorer differentiation [40]. For prognostication, Yoon et al. investigated 26 HER2 + gastric cancer patients before trastuzumab-treatment. In their analysis, they found GLCM contrast, variance, correlation and angular second moment (also known as energy or uniformity) were associated with a poorer survival [41]. In another study, Giganti et al. showed in 56 patients, that first-order energy, entropy, and skewness were significantly associated with a negative prognosis [42]. Giganti et al. also assessed pre chemotherapy texture features derived from the late arterial phase of 34 patients. They found entropy and compactness were higher and uniformity lower in responders [43]. No studies have assessed prognostication or response to therapy in gastric cancer.

## MRI radiomics

To date there have been little data for MRI in this tumor group as MRI is not performed routinely in the clinical pathway. There have been some exploratory data of pre-therapeutic ADC-maps of gastric cancer (Table 5). Liu et al. found that first-order statistics skewness may differ from node positive to node negative patients, and are associated with pathological characteristics including perineural and vascular invasion [44–47]. However, no studies so far have investigated prognostication or response assessment.

## Discussion

To date 22 imaging studies have been published investigating radiomic approaches in esophageal, esophagogastric, and gastric cancer, predominantly focused on texture analysis. Preliminary data for esophageal and esophago-gastric cancer suggest that there is potential for radiomic approaches in improving patient stratification for therapy. Eight 18F-FDG PET studies investigated the feasibility of heterogeneity analysis for response prediction (four studies with pre-therapy imaging only). Among the most often reported significant feature was GLCM entropy, reflecting the local randomness (irregularity) within the image, and where low GLCM entropy represents a more homogeneous texture. The reported accuracy for successful classification of therapy response ranged from 0.7 to 1.0 (AUC).

Nearly all published studies incorporated “classical” PET parameters e.g., SUV_max_, total lesion glycolysis and metabolic tumor volume into predictive models. In general radiomic parameters contributed to predictive models and provided additional information to standard parameters. Three CT studies of esophageal cancer have also suggested that greater tumoral heterogeneity is associated with poor response and outcome.

PET-studies investigating texture features as a prognosticator were more mixed. Only two studies found associations with overall-survival. The CT and MRI data for gastric cancer were also varied. Two studies found several features to be associated with survival time, however, for some parameters, e.g., histogram entropy and energy, it was surprising to find both parameters to be associated in the same direction given what they represent mathematically.

A challenge for interpretation of studies to date is the use of retrospective datasets with different imaging techniques across different scanners and/or institutions; different methodologies for feature selection; the focus on different feature sets; the lack of transparency in methodology with the use of different in-house software; as well as varying statistical and bioinformatics approaches for data analysis and interpretation. This has been highlighted by researchers in the field [48].

Moving forward in the context of esophageal and esophago-gastric cancer, it is important to improve our data quality. Planned prospective studies incorporating quality control is a step in the right direction to improving data curation and ensuring prediction models are fit for purpose and fulfill the promise of radiomics for improving patient stratification.

## Figures and Tables

**Table 1:** Overview of features used in radiomics

Feature-group	Parameter examples
First-order-histogram statistics	Mean, median, skewness, kurtosis, energy (uniformity), entropy
Second-order gray-level co-occurrence matrix (GLCM) statistics	Entropy, homogeneity, energy (uniformity), contrast, autocorrelation, cluster shade, cluster prominence, difference entropy, difference variance, dissimilarity, inverse difference moment, maximum probability, sum average, sum entropy, sum variance
Second-order gray-level difference matrix (GLDM) statistics	Mean, entropy, variance, contrast
High-order neighborhood gray-tone difference matrix (NGTDM) statistics	Coarseness, contrast, busyness, complexity, texture strength
High-order gray-level run-length (GLRL or RLM) statistics	Short run emphasis, long run emphasis, gray-level nonuniformity, run-length nonuniformity, intensity variability, run-length variability
High-order gray-level size zone matrix (GLSZM) statistics	Short-zone emphasis, long-zone emphasis, intensity nonuniformity, zone percentage, intensity variability, size zone variability
Fractal analysis	Mean fractal dimension, standard deviation, lacunarity, Hurst component

**Table 2:** Radiomic studies using PET in esophageal cancer

Author	PET time point	Therapy	Features assessed	Outcome and methods	Findings
Tixier et al. [26] *n* = 41 SCC:31 AC:10	Pre	CRT: 60 Gy with cisplatin or carboplatin/fluorouracil	38 features including: First order statistics GLCM RLM GLSZM NGTDM	Response prediction: AUROC	Tumor GLCM homogeneity, GLCM entropy, RLM intensity variability and GLSZM size zone variability can differentiate non-responders, partial responders, and complete-responders with higher sensitivity (76%–92%) than any SUV measurement
Beukinga et al. [27] *n* = 97 AC:88 SCC:9	Pre	CRT: 41.4 Gy with carboplatin/paclitaxel	88 features including: First order statistics Geometry GLCM NGTDM	Response prediction: Models constructed with least absolute shrinkage and selection operator regularized logistic regression	18F-FDG long run low gray level emphasis higher in responders than non-responders Model including histologic type, clinical T stage, 18F-FDG long run low gray level emphasis and non-contrast CT run percentage has higher AUC than SUV_max_: 0.74 vs. 0.54 (after internal validation)
Nakajo et al. [28] *n* = 52 SCC	Pre	CRT: 41–70 Gy with cisplatin/5-flurouracil	GLCM: Entropy, homogeneity, dissimilarity; GLSZM: Intensity variability, Size-zone variability, zone percentage	Response prediction: AUROC Prognostication: Multivariable cox analysis	GLSZM intensity variability and GLSZM size-zone variability predictive of response No texture parameter independently associated with progression free or overall survival
Paul et al. [29] *n* = 65 SCC:57 AC:8	Pre	CRT: 50 Gy with platinum chemotherapy & 5-flurouracil	84 features including: First order statistics GLCM GLSZM GLDM	Response prediction Prognostication: Multivariable cox analysis	Best subset of predictive variables: metabolic tumor volume, GLCM homogeneity Best subset of prognostic variables: WHO performance status, nutritional risk index, metabolic tumor volume
Foley et al. [17] *n* = 403 AC:237 + 79 SCC:65 + 22	Pre	NACT, NACRT, dCRT: not specified	First order statistics GLCM: homogeneity, entropy, dissimilarity; coarseness; GLSZM: intensity variability, large area emphasis, zone percentage;	Prognostication: Multivariable cox analysis	TLG, histogram energy and histogram kurtosis are independently associated with overall survival
Tan et al. [31] *n* = 20 AC:17 SCC:3	Pre-post	CRT: 50.4 Gy with cisplatin/fluorouracil	192 features including: First order statistics GLCM	Response prediction: AUROC	SUV_mean_ decline, SUV skewness, GLCM inertia, GLCM correlation, and GLCM cluster prominence are predictors of complete response with AUC 0.76–0.85
Van Rossum et al. [32] *n* = 217 AC	Pre-post	CRT: 45 or 50.4 Gy with fluoropyrimidine and either a platinum compound or taxane	86 features including: First order statistics Geometry GLCM NGTDM	Response prediction: Multivariable Cox analysis	Feature selection by univariable logistic regression Model including induction chemotherapy, change in RLM run percentage, change in GLCM entropy, and post –CRT roundness is better than clinical prediction model
Yip et al. [33] *n* = 45 AC:44 SCC:1	Pre-post	CRT: 45–50.4 Gy with cisplatin, 5-flurouracil, irinotecan/paclitaxel or carboplatin/paclitaxel	GLCM: homogeneity, entropy RLM: high gray run emphasis, short-run high gray run emphasis GLSZM: high gray zone emphasis, shortzone high gray emphasis	Response prediction: AUROC	Response prediction: Change in run length and size zone matrix parameters differentiates non-responders from artial/complete responders (AUC: 0.71–0.76)
Beukinga et al. [33] *n* = 70 AC:65 SCC:8	Pre-post	CRT: 41.4 Gy in 23 fractions with carboplatin/paclitaxel	113 features including: First order statistics Geometry Local intensity GLCM GLSZM NGTDM	Response prediction: Models constructed with least absolute shrinkage and selection operator regularized logistic regression	Prediction model composed of clinical T-stage and post-NCRT joint maximum adds important information to the visual PET/CT evaluation of a pathologic complete response

Studies are ordered after time point of imaging (pre therapy, or pre-post therapy imaging), evaluated outcome (response prediction, prognostication, or both), and finally chronologically *dCRT* definitive chemoradiation; *NACT* neoadjuvant chemotherapy; *NACRT* neoadjuvant chemoradiation; *GLCM* gray-level co-occurrence matrix; *GLDM* gray-level difference matrix, *GLSZM* gray-level size zone matrix; *NGTDM* neighborhood gray tone difference matrix; *RLM* gray-level run-length matrix; *AUROC* area under the receiver operative curve; *AUC* area under the curve

**Table 3:** Radiomic studies using CT in esophageal cancer

Author	CT time point & type	Therapy	Features assessed	Outcome and methods	Findings
Hou et al. [35] *n* = 49 SCC	Pre Contrast enhanced	CRT: 60 Gy with nedaplatin/docetaxel or nedaplatin/paclitaxel	214 features including: First order statistics Geometry GLCM RLM GLSZM NGTDM with Gabor transformation or Gaussian filtration	Response prediction Feature selection: AUROC Prediction model: support vector machine and artificial neural network	Features discriminating non-responders from responders: skewness, kurtosis, GLSZM long zone emphasis, Gabor_MSA-54, Gabor2D_MSE-54
Ganeshan et al. [36] *n* = 21 AC:14 SCC:7	Pre Non-contrast from PET/CT	No information available	First order statistics with Gaussian filtration	Prognostication: Kaplan–Meier analysis	High uniformity is an independent predictor of survival. Lower uniformity is associated with a poorer overall survival
Yip C. et al. [37] *n* = 36 AC:9 SCC:26	Pre-post Contrast enhanced Arterial Portal venous	CRT: 50 Gy with cisplatin/5-flurouracil or single agent platinum/5-flurouracil	First order statistics with Gaussian filtration	Prognostication: Kaplan-Meier analysis Cox analysis	Higher post treatment entropy (medium/coarse) independently associated with poorer overall survival

*CRT* neoadjuvant chemo-radiotherapy; *NACT* neoadjuvant chemotherapy; *AC* adenocarcinoma; *SCC* squamous cell carcinoma; *Th* thorax; *Ab* abdomen; *RLM* gray-level run-length matrix; *GLCM* gray-level co-occurrence matrix; *GLSZM* gray-level size zone matrix; *NGTDM* neighborhood gray tone difference matrix; *OS* overall survival

**Table 4:** Radiomic studies using CT in gastric cancer

Author	CT time point & type	Therapy	Features assessed	Outcome and method	Findings
Ba-Ssalamah et al. [38] *n* = 67 (Art) AC:47 GIST:15 Lymphoma:5 *n* = 73 (PV) AC:48 GIST:17 Lymphoma:5	Pre Contrast enhanced Arterial Portal venous	Not applicable	First order statistics RLM GLCM Absolute gradient Autoregressive model, wavelet transformation	Classification: linear discriminant analysis	Classification of lymphoma vs. AC or GIST feasible on arterial phase: AC vs. lymphoma: 3.1% misclassification GIST vs. lymphoma: 0% misclassification on arterial CT Classification of AC vs. GIST feasible on venous phase: 10% misclassification
Ma et al. [39] *n* = 70 AC:40 Lymphoma:30	Pre Contrast enhanced Portal venous	Not applicable	First order statistics Geometry Texture analysis Wavelet transformation	Feature selection: LASSO Classification: AUROC	183 radiomic signature identified with potential to differentiate adenocarcinoma from lymphoma Model including histogram root mean squared, GLCM sum variance and absence of peristalsis: AUC 0.86; Sensitivity 70%, Specificity 100%, Accuracy 87%
Liu et al. [40] *n* = 107 AC:84 Signet Ring:5 Mucinous:3 Mixed:15	Pre Arterial Portal venous	Surgery	First order statistics	Classification: AUROC	Arterial phase standard deviation and entropy; portal venous phase mean, max, mode, percentiles are predictive of poorer differentiation
Yoon et al. [41] *n* = 26 AC:25 Signet Ring:1	Pre Portal venous	Trastuzumab-based chemotherapy	First order statistics: Kurtosis, Skewness GLCM: Angular second moment, contrast, entropy, variance, correlation	Prognostication: AUROC Kaplan-Meier	Lower contrast, variance and higher correlation are associated with poorer survival with AUC of 0.77, 0.75 and 0.77 respectively
Giganti et al. [42] *n* = 56 AC:37 Signet Ring:19	Pre Arterial	Surgery	107 features including: First order statistics GLCM RLM Geometry with Gaussian filtration	Prognostication: Kaplan-Meier, Multivariable Cox analysis	Energy, entropy, skewness are associated with poorer prognosis
Giganti et al. [43] *n* = 34 AC:25 Signet Ring:6	Pre Arterial	NACT: cisplatin/epirubicin/adriamycin/fluoruracil or cisplatin/epirubicin/aadriamycin/capecitabine	First order statistics GLCM Geometry	Response prediction: Multivariable logistic model	Entropy and compactness are higher in responders and uniformity is lower in responders

*AC* adenocarcinoma; *GIST* gastrointestinal stromal tumor; *NACT* neoadjuvant chemotherapy; *RLM* gray-level run-length matrix; *GLCM* gray-level co-occurrence matrix; *AUROC* area under the receiver operative curve

**Table 5:** Radiomic studies using MRI in gastric cancer

Author	MRI time point & sequence	Therapy	Features assessed	Outcome and methods	Findings
Liu et al. [44]^a^ *n* = 80 AC:57 Signet ring:10 Mucinous:1 Mixed:12	Pre ADC-map	Surgery	First order statistics	Staging: Prediction of tumor & nodal stage: AUROC analysis	ADC histogram analysis may differentiate node positive from node negative disease e.g., Percentile ADC_10_ has an AUC of 0.79 and sensitivity, specificity and accuracy of 72%, 81% and 74% respectively No ability to differentiate T stage
Liu et al. [45]^a^ *n* = 87 AC:60 Signet ring:11 Mucinous:1 Mixed:15	Pre ADC-map	Surgery	First order statistics	Staging: Prediction of tumor & nodal stage: AUROC analysis	Skewness yields a sensitivity and specificity of 76% and 81%, and an AUC of 0.80 for differentiating node positive from node negative gastric cancers
Zhang et al. [47]^a^ *n* = 78 AC:58 Signet ring:11 Mucinous:1 Mixed:8	Pre ADC-map	Surgery	First order statistics	Classification: AUROC analysis	ADC histogram parameters differ between histological grades but with an AUROC < 0.70 this may not be useful in clinical practice
Liu et al. [46]^a^ *n* = 64 AC:45 Signet ring:8 Mucinous:2 Mixed:9	Pre ADC-map	Surgery	First-order Entropy GLCM Entropy	Classification (Grade): AUROC analysis	First-order entropy may differentiate between gastric cancers with vascular invasion with a sensitivity, specificity, accuracy of 86%, 75%, 81% and AUC of 0.82

*AC* adenocarcinoma; *AUROC* area under the receiver operative curve; *GLCM* gray-level co-occurrence matrix

a Same institution data
